# The Benefits of Physical Exercise on Mental Disorders and Quality of Life in Substance Use Disorders Patients. Systematic Review and Meta-Analysis

**DOI:** 10.3390/ijerph17103680

**Published:** 2020-05-23

**Authors:** Jorge Giménez-Meseguer, Juan Tortosa-Martínez, Juan M. Cortell-Tormo

**Affiliations:** Faculty of Education, University of Alicante, 03690 San Vicente del Raspeig, Spain; jgimenezmeseguer@gmail.com (J.G.-M.); jm.cortell@gcloud.ua.es (J.M.C.-T.)

**Keywords:** substance use disorders, drug addiction, physical exercise, physical activity, quality of life, mental disorders, anxiety, depression, stress, craving

## Abstract

Physical exercise seems to have a promising effect on numerous variables related to the recovery of drug-dependent patients. However, some contradictions are found in the literature. The aim of this study was to perform a systematic review and meta-analysis in order to identify the effect of physical exercise on mental disorders, quality of life, abstinence, and craving, and make a comparison of the effect of exercise depending on the type of program. A search for articles was conducted using PubMed, Web of Science, and Scopus databases. Studies were selected that measured the acute effects or long-term effect (≥2 weeks) of exercise in patients who met criteria for alcohol use disorders or substance use disorders. A total of 59 studies were included. An effect of exercise on mental disorders (standardized mean differences (SMD) = 0.66 (confidence interval (CI): 0.46, 0.86); z = 6.50; *p* < 0.00001) and quality of life (SMD = 0.69 (95% CI: 0.53, 0.84); z = 8.65; *p* < 0.00001) was identified. Subgroup analysis revealed an effect of exercise in craving (SMD = 0.80 (CI: 0.07, 1.53); z = 2.15, *p* = 0.03), stress (SMD = 1.11 (CI: 0.31, 1.91); = 2.73; *p* = 0.006), anxiety (SMD = 0.50 (CI: 0.16, 0.84); z = 2.88; *p* = 0.004) and depression (SMD = 0.63 (CI: 0.34, 0.92); z = 4.31; *p* < 0.0001). Body–mind activities and programs based on improving physical conditions produced similar results in mental disorders and quality of life. Available evidence indicates that physical exercise, both body–mind and physical fitness programs, can be effective in improving mental disorders, craving, and quality of life in drug-dependent patients.

## 1. Introduction

Drug use is a worldwide problem that persists throughout the years. According to the World Drug Report (2016) about 250 million people between 15 and 64 years tried some type of drug in the year 2014. Among them, one in ten was a “problematic consumer”, resulting in physical, psychological and/or social problems. Therefore, approximately 30 million people are suffering from problems derived from drug use (diseases, psychiatric disorders, physical deterioration, social exclusion, family problems, emotional disorders, etc.) [1].

The consequences of drug use include a serious deterioration in quality of life and mental health. This means that drug addiction rehabilitation treatments should not only be focused on achieving mere abstinence, but should also try to recover the quality of life of the patient and their physical and mental health [2].

Given this problem, performing physical exercise can be an effective tool to help in the prevention and treatment of drug dependencies at different levels. On one hand, physical exercise seems to be a preventative factor for the consumption of drugs. People who practice physical exercise on a regular basis have a lower rate of drug use problems and vice versa [3].

On the other hand, physical exercise can be equally effective for the treatment of drug addictions. Thus, there have been lower rates of consumption and/or “craving” in consumers of alcohol [4,5,6], cannabis [7], cocaine [8,9], methamphetamine [10,11,12] or other substances [13,14] after participation in physical exercise programs.

In addition to the effect on consumption and craving, exercise has also been proven effective as a method to improve mental disorders such as stress reduction [15], anxiety [16,17] and depression [16,18]. The DSM-5 defines a mental disorder as “a syndrome characterized by clinically significant disturbance in an individual’s cognition, emotion regulation, or behavior that reflects a dysfunction in the psychological, biological, or developmental processes underlying mental functioning.” As a consequence, there is usually a distress or impairment in personal, family, social, educational, occupational, or other important areas of functioning [19].

Likewise, some studies have observed that a program combining aerobic exercise with strength training can improve the quality of life in a drug-dependent population [6,20] and that yoga can be an equally valid tool for improving the quality of life [21]. In the same line, recent studies describe the influence that physical exercise can have at a cognitive level [22] and even in satisfaction of life [23]. Quality of life may be defined as “an individual’s perception of their position in life in the context of the culture and value systems in which they live and in relation to their goals, expectations, standards and concerns. It is a broad ranging concept affected in a complex way by the person’s physical health, psychological state, personal beliefs, social relationships and their relationship to salient features of their environment” [24].

However, benefits are not always obtained after participation in a physical exercise program, as recent studies show. For example, a varied physical exercise program of 12 weeks in heroin addicts undergoing rehabilitation was not proven to be effective to obtain significant benefits in stress, anxiety, and quality of life [25]. Therefore, it is likely that the type of exercise program used and the structural parameters of the exercise (type of activity, intensity, volume, or frequency) can play a key role in the effects obtained after participating in a physical exercise program.

Analyzing the literature, there are studies that have achieved positive effects using an aerobic exercise program [5,7,8,26], others that reach similar goals with a combined program of aerobic exercise or strength [6,20,27], some who have used a high-intensity interval training program [18] or others who have used activities related to meditation or relaxation, such as yoga, tai-chi or qigong with satisfactory results [9,21,28,29,30,31].

In this way, generalizing about the effectiveness of the practice of physical exercise in the treatment of drug dependence can be complex, since the studies carried out employ different modalities of physical activity/physical exercise and different intensities, volumes and frequencies.

For this reason, the production of a review and meta-analysis of studies that investigate the influence of physical exercise in the population with substance use disorders (SUD) is of special importance, taking into account the different types of programs used, and identifying from a broad and global perspective the different benefits observed after participating in one exercise program or another.

In recent years, some review studies have been published trying to identify the benefits of physical exercise in participants who met the criteria for alcohol use disorders (AUD) or other substances use disorders [32,33,34,35,36,37,38] at different levels. Some meta-analysis have also been carried out with the same objective, focusing on SUD patients [39] and AUD patients [34]. These meta-analysis found, on the one hand, that physical exercise can increase the abstinence rate and reduce withdrawal symptoms, anxiety, and depression symptoms in SUD patients [39]. On the other hand, Hallgren and colleagues found that exercise can improve fitness and reduce depression symptoms in AUD patients [34].

However, to the best of our knowledge, there is no meta-analysis focusing on the benefits of exercise on quality of life, stress or craving in drug-dependent patients.

There are also very few studies that compare the effectiveness of one type of physical exercise/physical activity or another in terms of the benefits obtained. Aerobic exercise is the most commonly used type of exercise for drug addicts [34,39]. However, body–mind activities are increasingly used, such as yoga, tai chi, or qigong [33]. Both practices seem to be beneficial for the treatment of drug dependence, but it is unknown which of the two can generate greater benefits, and whether the use of aerobic exercise or body–mind practices may be more appropriate for aspects of mental disorders and quality of life. In this sense, Wang and colleagues [39] compared aerobic exercise with “mind–body exercise”, concluding that both activities are effective for the improvement of abstinence, the symptoms of withdrawal syndrome, anxiety and depression, without appreciable differences between one type of activity and another. In the same way, they did not find differences regarding the type of intensity used. However, further research is required to identify the differences between types of exercise as well as the optimal intensity, frequency or duration of the programs, especially for the quality of life of this population.

Therefore, the objective of this review and meta-analysis was to perform an updated analysis of the benefits of physical exercise in AUD or SUD patients, quantify the effect of physical exercise on mental disorders, craving, and quality of life, and compare the effect of exercise programs with the most commonly used body–mind practices.

## 2. Method

This study follows the guidelines of the Preferred Reporting Items for Systematic reviews and Meta-Analysis (PRISMA) [40] and the Meta-Analysis and Systematic Reviews of Observational Studies (MOOSE) [41].

### 2.1. Research Strategies

For the selection of articles, a systematic search was made of articles published in PubMed, Web of Science and Scopus databases. Combinations of the key words “exercise, physical activity, qigong, taichi, yoga, substance use disorders, substance abuse, drug abuse, cocaine, cannabis, marijuana, alcohol, heroin, amphetamine, hypnotics, inhalants, hallucinogens, mdma, methamphetamine” were used.

The bibliographic references of the studies found were also examined manually to obtain new references. All of the identified articles were independently analyzed by two investigators of the present study, and the final selection of the articles to be included was taken by both, in a consensual manner.

We contacted some authors of the identified studies to ask for additional information or clarify some data for the meta-analysis.

### 2.2. Selection of Studies

Studies were selected that measured the acute effect of exercise (a single session) or experimental or quasi-experimental studies that measured the long-term effect of exercise in AUD or SUD patients. The studies that employed body–mind activities such as tai chi, yoga, or qigong, were also included.

Studies that fulfilled any of the following characteristics were discarded: studies conducted with animals, patients under 18 years of age, studies that exclusively analyzed tobacco addiction, review studies, observational studies or those that do not apply any program of physical exercise, and case studies. For the meta-analysis, studies without defined relevant statistical data, whose authors could not be contacted or did not provide the data when contacted, were also discarded.

**Figure 1:**
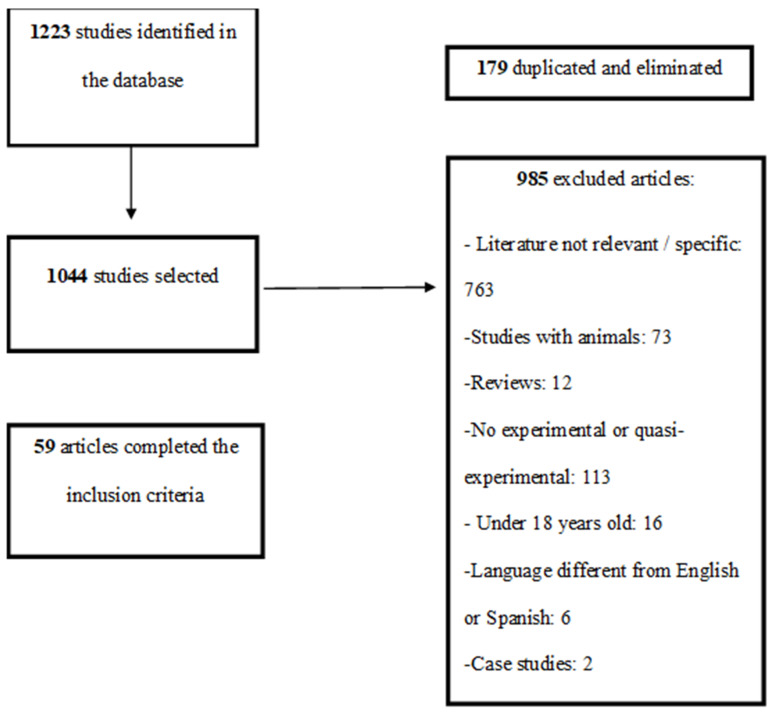
Flowchart of the study selection process.

All the studies addressed the analysis of any of the following variables: physical condition/fitness/physiological markers, quality of life, stress, anxiety, depression, craving, abstinence/indications of consumption, other psychiatric or psychological variables. Figure 1 shows the process followed from the initial search to the final selection of the studies.

### 2.3. Data Extraction

The studies were thoroughly analyzed and the relevant data were collected in a table that contained the following categories: Population/substance of consumption, sample (experimental + control), type of exercise program, adherence to the program, measured variables and main results obtained.

### 2.4. Quality Analysis of the Studies

To define the quality of the studies, the widely used and validated “Physiotherapy Evidence Database (PEDro)” scale was taken into account [42,43,44]. This scale is based on the Delphi list [45], but it adds two criteria (items 8 and 10) that are not included in the Delphi list and are important when analyzing the quality of the studies. Of the 11 items on the PEDro scale, 5 and 6 were discarded for the analysis of the studies, as did other similar previous studies, among them, Wang and colleagues [39], since the blinding of patients and the instructors regarding the type of treatment used could not be applied in the studies that were contemplated.

In each study, it was analyzed if they met each of the nine criteria or not, adding one point for each criterion met and zero points for each criterion not met or not specified, with 9 points being the highest possible score and 0 points the minimum.

### 2.5. Meta-Analysis

For the meta-analysis, standardized mean differences (SMD) were calculated, with the respective 95% confidence interval (CI95%). The value of statistical significance was established at p < 0.05. On the other hand, the following criteria of statistical heterogeneity (I2) were established: 0–40% mild, 40–70% moderate and 70–100% high. A random effects model was selected when the heterogeneity values were adequate (I2 > 50%). Difference from post-intervention control group to post-intervention experimental group was calculated. In addition, the difference in the post-intervention control to post-intervention experimental change scores between participants in programs based on improving physical conditions and those based on oriental practices. A subgroup analysis was performed, according to the values of quality of life, mental health, and according to the physical activity modality investigated, for which effect estimates were obtained with the analysis model described above. The software proposed by Cochrane, Review Manager 5.3 (RevMan) was used to calculate the effect estimates.

To test for possible publication bias, we used the trim-and-fill funnel plot derived method, which identifies possible publication bias by estimating the number of unpublished studies and adjusts results for this bias. Meta-Essentials tool was used to build funnel plots and to statistically test for funnel plot asymmetry with the Trim and Fill test.

## 3. Results

### 3.1. Descriptive Characteristics of the Selected Studies

In total, 59 studies are included, with a total of 3792 participants. Of the 59 studies, 52 measured the long-term effect of exercise [4,5,6,7,8,9,12,13,14,15,16,17,18,20,21,22,25,26,27,28,29,30,46,47,48,49,50,51,52,53,54,55,56,57,58,59,60,61,62,63,64,65,66,67,68,69,70,71,72,73,74,75,76], with exercise programs ranging from two weeks [7,65] to 12 months [6]. The duration of the most common exercise programs was 12 weeks. Samples of the studies range from seven participants [57] to 218 participants [75]. Seven studies measured the acute effect of exercise [5,10,11,77,78,79,80]. 

Of the 59 studies, 24 include only AUD patients [4,5,6,29,46,47,48,49,50,51,52,53,54,55,56,57,58,59,60,61,77,78,79,80], 15 include patients who met criteria for different substances disorders, describing groups among which are AUD or SUD patients [13,14,16,18,20,26,28,62,63,64,66,67,70,72], eight studies include methamphetamine dependence patients [10,11,12,17,27,68,73,76], five studies include heroin or opioids dependence patients [21,25,30,65,71], four studies include cocaine or “crack” dependence [8,9,15,22], two studies include stimulants dependence patients, including patients who met criteria for cocaine, amphetamines or methamphetamines dependence [74,75], and one study includes cannabis dependence patients [7].

Of the selected studies, 34 were experimental studies with randomized sampling [4,5,8,9,11,12,15,17,18,21,25,27,29,30,46,48,49,52,54,55,59,60,61,63,64,65,68,69,70,71,74,75,76,77], while the rest were quasi-experimental [6,7,10,13,14,16,20,22,26,28,47,50,51,53,56,57,58,62,66,67,72,73,78,79,80]. 

Aerobic exercise was the most used type of program, finding 27 studies that used this type of exercise [4,5,7,8,10,11,12,13,16,18,22,26,46,49,50,56,57,59,60,61,67,74,75,76,77,78,79]. In addition, 14 studies were found that used exercise programs related to body–mind practices, such as yoga, tai chi or qigong [9,15,21,28,29,30,54,55,64,65,66,70,71,73], 10 who used combined strength and aerobic exercise [6,17,20,27,32,47,48,52,68,72], five that included varied physical activities [14,25,53,58,69], one that used a strength program [80], one that used softball [62] and finally, a study that compared the effects of three different exercise programs (aerobic, strength, and circuit training) [63].

### 3.2. Methodological Quality of the Selected Studies

Table 1 shows the analysis of the quality of the included studies, finding scores ranging from 1 point to 8 points. The average score of the studies was 4.97 (±1.85) out of 9. Several of the studies did not provide enough information to understand if some of the criteria were met or not. In these cases, the criterion was given as not fulfilled.

**Figure 2:**
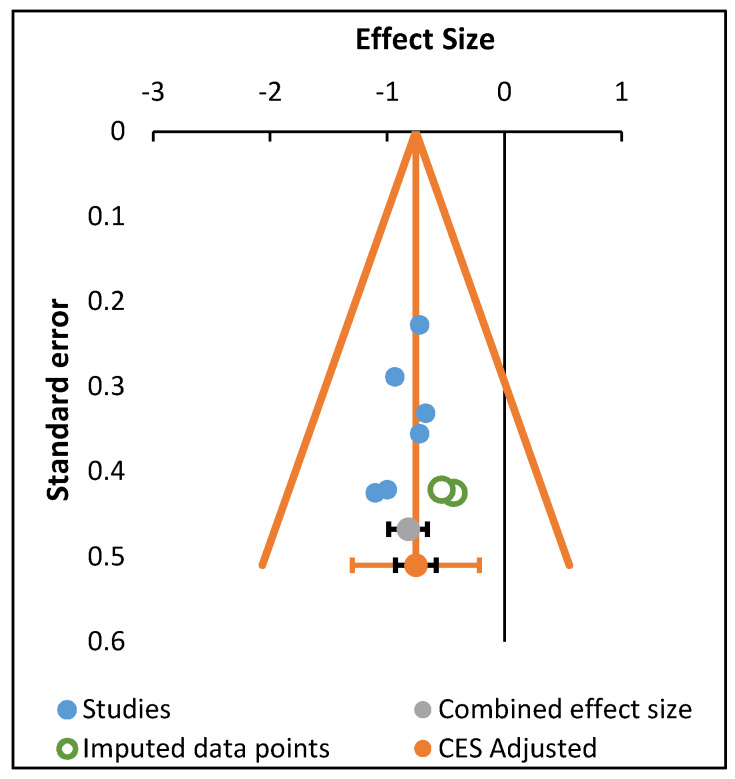
Trim and Fill funnel plot (Role Emotional).

**Figure 3:**
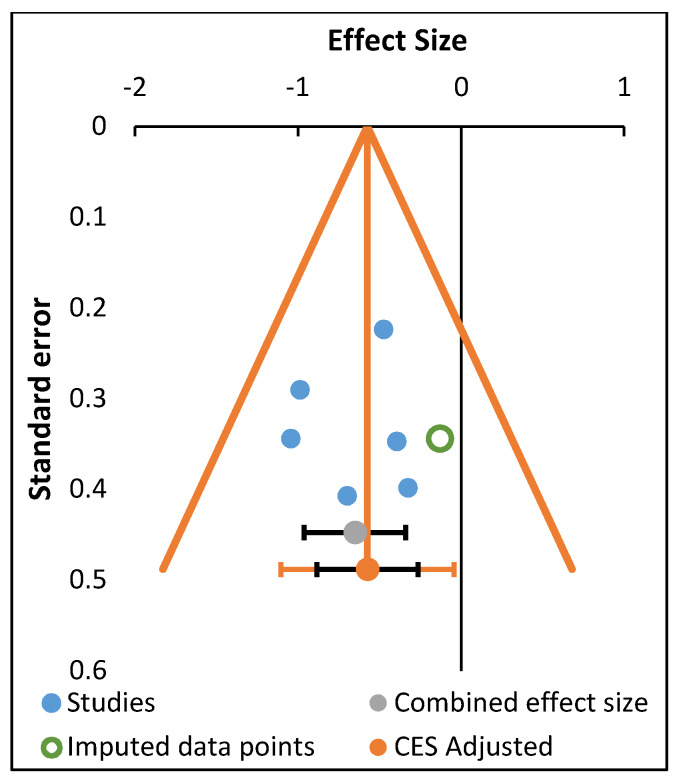
Trim and Fill funnel plot (Mental Health).

Based on the Trim and Fill test, the lack of imputed data points in the funnel plots indicates the absence of asymmetry in the distribution of effect sizes when considering the effects of all programs together, the physical fitness programs and oriental practices on mental disorders and craving. Asymmetries were only found when considering the effect of all programs on quality of life, especially on Role Emotional (two missing studies) and Mental Health (one missing study) (see Figure 2; Figure 3, respectively).

### 3.3. Acute Effect of the Exercise

Seven studies were found that analyzed the acute effect of exercise, five of them in AUD patients, in which inconsistent results were obtained on the effect of exercise in this population [5,77,78,79,80] and two in methamphetamine dependents, where an effect of acute exercise on craving is shown [10,11]. In all of them, an aerobic exercise session was performed, except for one [80], which performed a strength session. The results are summarized in Table 2.

### 3.4. Long-Term Effect of Exercise

**Benefits of exercise on physical conditions**

Of the 59 studies included in the present study, 24 measured the effect of a physical exercise program on physical conditions [4,8,13,16,18,22,26,27,46,47,48,49,50,51,52,53,56,57,58,60,63,67,68,72]. All of them used pure aerobic exercise programs or combined with strength exercises. Of the 24, all showed improvements in physical conditions after the end of the program, except for five studies [4,51,57,60,63].

**Benefits for the quality of life**

This review includes 10 studies that specifically analyze the influence of exercise on quality of life: five of them use yoga as an experimental treatment [15,21,55,70,71] a study uses tai chi [73], two studies apply a program of mixed exercise and aerobic exercise [6,72] and another study employs a varied exercise program [25].

Of the 10 studies, eight obtained significant improvements in quality of life after participation in the program [6,20,21,55,70,71,72,73], while in two studies there were no significant improvements [15,25].

**Benefits for mental disorders**

A total of 23 studies are included that analyze the influence of exercise on stress, anxiety and/or depression. Of these 23 studies, six studies assess the effect of exercise on stress. Two studies showed improvements in the levels of this variable after participation in a yoga program [15,55], while there are four studies where, despite showing better levels after the end of the program, these improvements were not significant in comparison with the control group, both in yoga programs [29,66], and in varied exercise programs [25,69].

In anxiety, six studies were found that showed statistically significant benefits after participation in programs of aerobic exercise [16,51], strength [63], mix of aerobic exercise and strength [17], yoga [21] or qigong [65], while in seven other studies, anxiety levels were improved, but this improvement was not statistically significant or was only intra-group, both in yoga programs [28,29] and in aerobic exercise programs [4,18,57] or mixed [20,51].

Finally, we found eight studies that show a statistically significant improvement in depression after participation in aerobic exercise programs [16,51], strength [63], mixed [17], yoga [21,54,70] or qigong [9]. On the other hand, another eight studies were found that, in spite of improving the levels, did not show statistically significant improvements compared to the control group after participation in aerobic exercise programs [4,18,57], mixed [21], yoga [28,29], tai chi [30] or combined programs of yoga with aerobic work [53].

**Benefits for abstinence and craving**

We found 25 studies that analyzed the effect of exercise on aspects related to abstinence/consumption and/or the impulse to consume (craving). Among them, 17 studies showed statistically significant improvements in abstinence [4,7,13,14,50,56,58,62,65,74,75] and in craving [5,7,9,12,28,53], whereas seven studies did not find statistically significant improvements, or these improvements were intra-group but not between groups, both in abstinence [8,16,29,52,59,60,69] and craving [8].

The following tables show the results and characteristics of the studies conducted with patients addicted to alcohol (Table 3) or other drugs (Table 4).

### 3.5. Meta-Analysis

**Quality of life**

**Figure 4:**
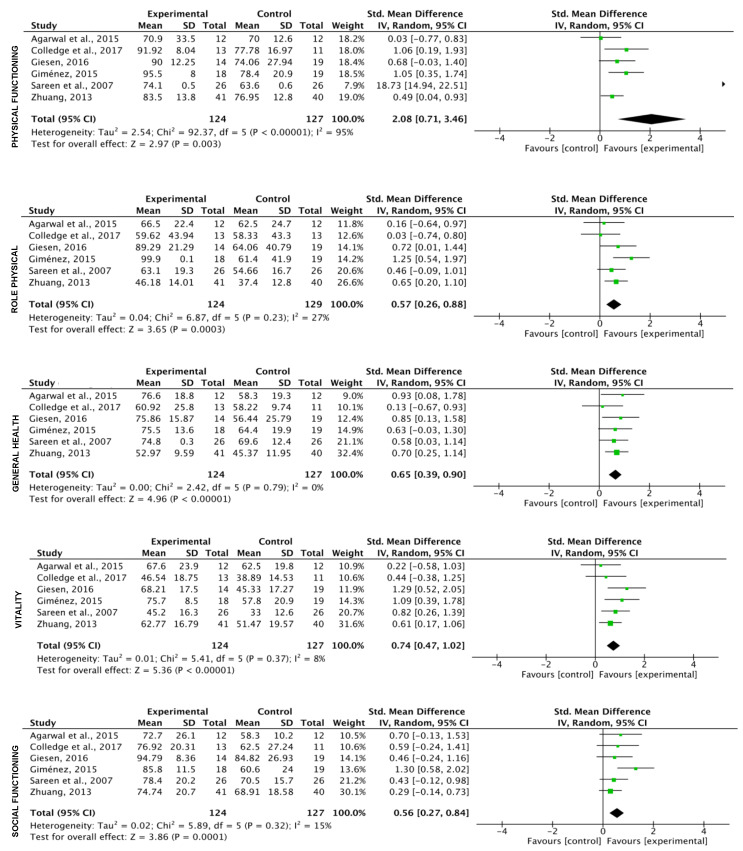

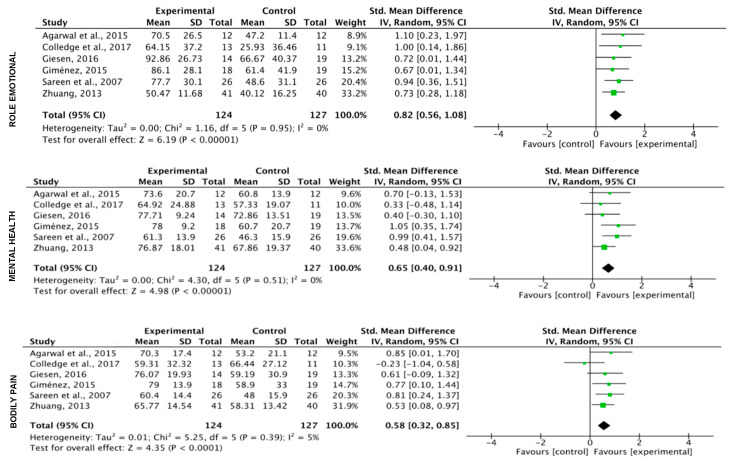
Effect of the exercise on quality of life (all programs included).

The results of the analysis showed a significant effect of physical exercise (k = 6) in the eight variables included in the SF36 test (Figure 4). The Trim and Fill adjusted the values only for Role Emotional (SMD = −0.76 (CI: −0.93, −0.58); Z = −12.78; *p* = 0.000) and Mental Health (SMD = −0.57 (CI: −0.88, −0.26) Z = −5.37; *p* = 0.000).

**Mental disorders and craving**

The results show a significant effect of exercise (including all exercise programs here) on depression (k = 10; SMD = 0.63 (CI: 0.34, 0.92); z = 4.31; *p* < 0.0001), anxiety (k = 8; SMD = 0.50) CI: 0.16, 0.84); z = 2.88; *p* = 0.004) and stress (k = 4; SMD = 1.11 (CI: 0.31, 1.91); z = 2.73; *p* = 0.006).

**Figure 5:**
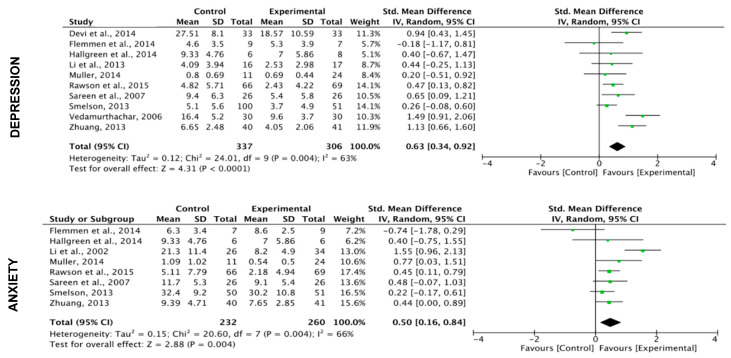

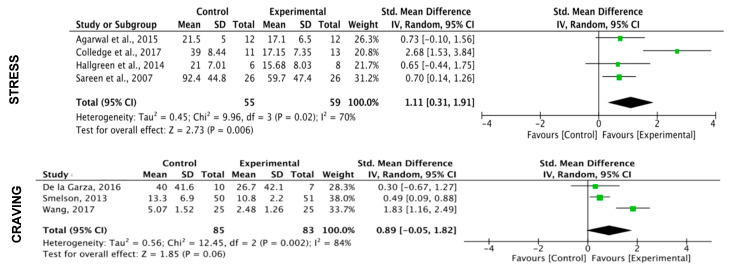
Effects of the exercise on mental disorders and craving (all programs included).

The results show also a significant effect of the exercise on craving (k = 3; SMD = 0.80 (CI: 0.07, 1.53); z = 2.15; *p* = 0.03). (Figure 5)

**Comparison between the effect of physical fitness (PF) programs and programs based on oriental practices (OP)**

**Quality of life**

**Figure 6:**
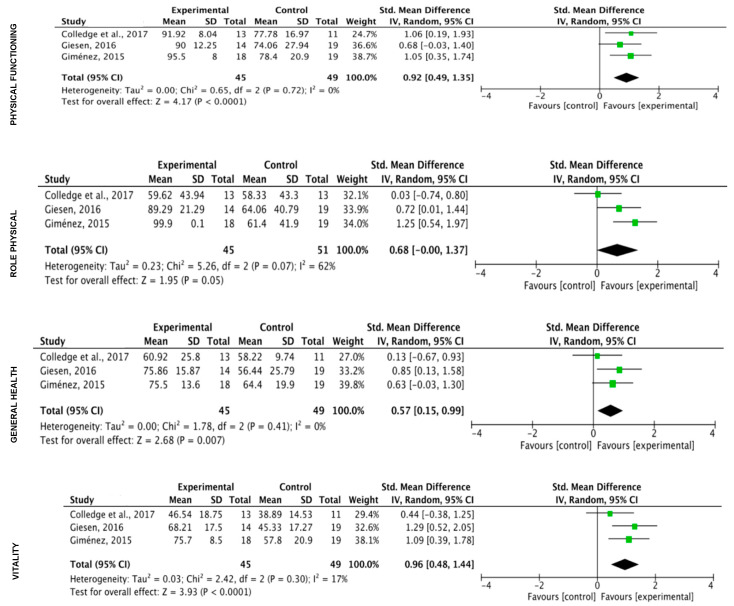

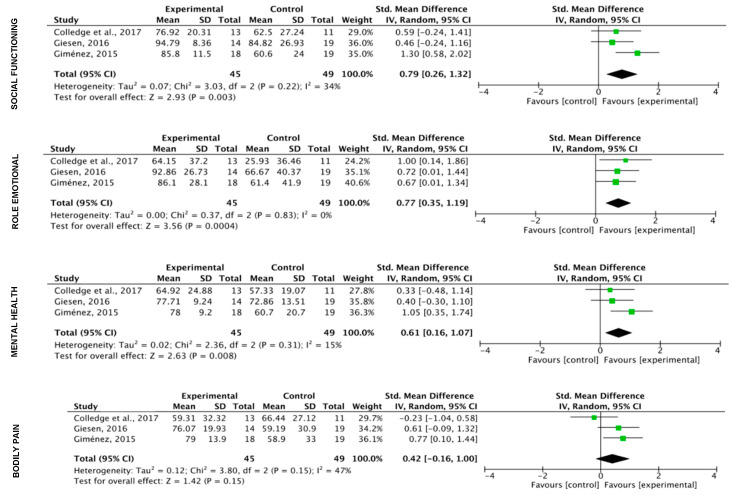
Effect of physical fitness programs on quality of life.

**Figure 7:**
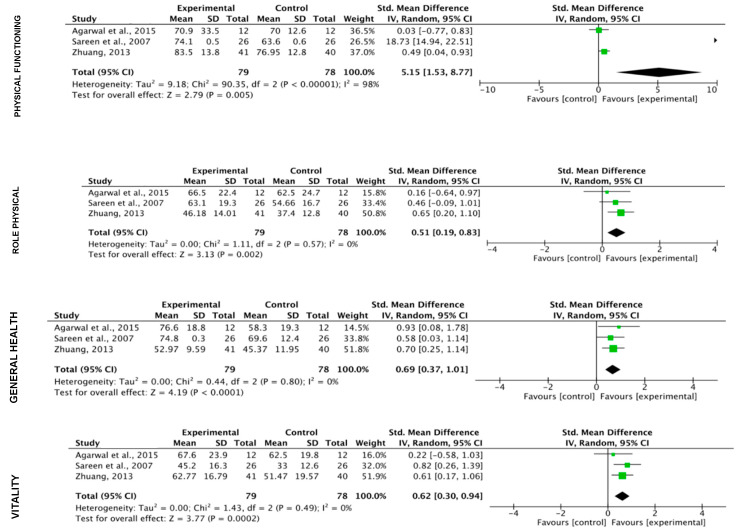

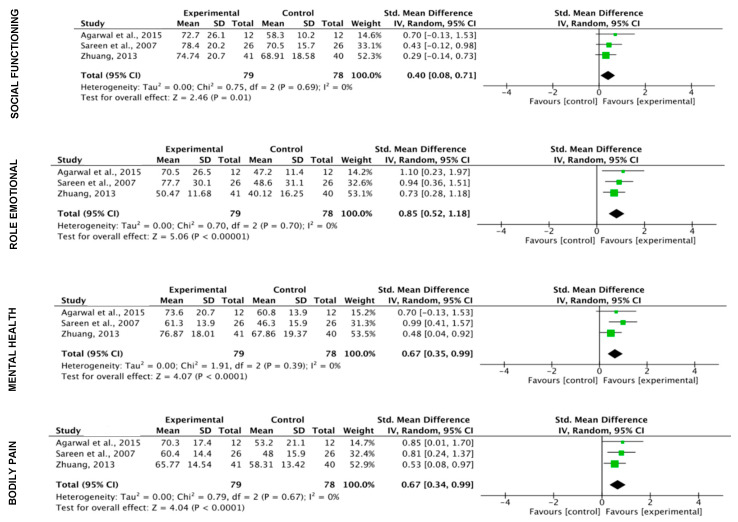
Effect of programs based on oriental practices on quality of life.

When analyzing the subcategories of the SF-36, a greater effect of OP (k = 3) is observed in the variables physical function and pain, a greater effect of PF (k = 3) in the variables “physical role”, “vitality” and “social function” and an similar effect in the variables “general health”, “emotional role” and “mental health” (Figure 6; Figure 7).

**Mental disorders and craving**

When analyzing the subcategories of mental disorders, a greater effect of OP is observed in the variables “anxiety” and “depression” and a greater effect of PF in the variable “stress”.

**Figure 8:**
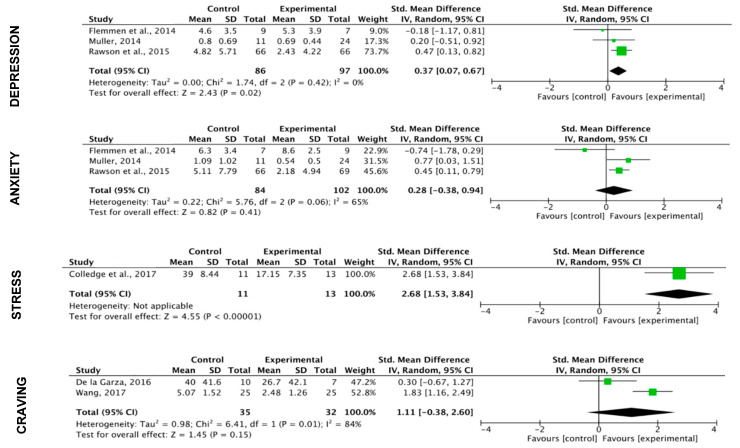
Effect of physical fitness programs on mental disorders and craving.

**Figure 9:**
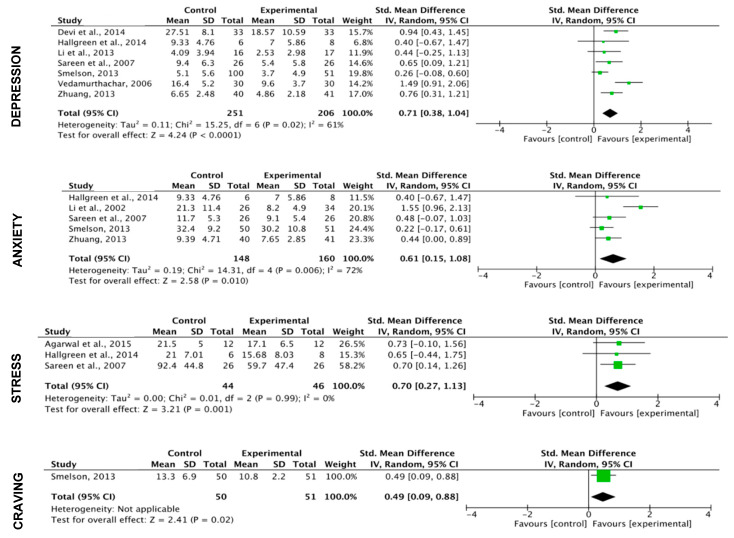
Effect of oriental practices on mental disorders and craving.

A non-significant effect of PF programs (k = 2; SMD = 0.92 (CI: −0.12, 1.96); z = 1.73; *p* = 0.08) and a significant effect of the OP programs (k = 1; SMD = 0.49 (CI: 0.09, 0.88); z = 2.41; *p* = 0.02) on craving was identified (Figure 8; Figure 9).

## 4. Discussion

The present study shows important results about the benefits of physical exercise in numerous variables related to health and quality of life in drug-dependent persons. The results indicated that physical exercise can be a good tool to improve physical conditions, mental health, quality of life and craving in drug-dependent persons and that different physical exercise practices produce similar results.

**Physical fitness**

First, the results show that the practice of aerobic exercise or combinations of aerobic exercise and strength allows patients to improve their physical conditions. Other review studies conducted with AUD patients came to the same conclusion [34,81].

Drug-dependent patients usually show deterioration in their physical health, either due to direct drug consumption, loss of healthy habits or both circumstances [26]. This makes drug-dependent patients more likely to develop diseases, such as metabolic disorders [82] or cardiac pathologies [83]. These types of pathologies are highly related to the level of physical condition [34]. Thus, an improvement in the physical condition of drug-dependent patients may potentially result in an improvement in their health with a decrease in the risk of developing comorbidities associated with drug use, highlighting the importance of improving physical conditions in this population.

However, the “improvement of physical condition” is something very generic, and to this day it is not clear which aspects of physical condition are more effective. Practically, all of the studies included in this review employ aerobic exercise programs, expressing the improvements in physical conditions expressed in terms of VO2, heart rate or performance in indirect tests that measure aerobic capacity. Some studies employ combinations of aerobic and strength exercise, while studies employing only strength or flexibility training are practically nil. In this sense, it would be convenient to carry out studies that also investigate the benefits that exercise programs may have for other physical abilities, such as strength and/or flexibility.

**Quality of life**

One of the novelties of the present study is the analysis performed in regards the effect of exercise on the quality of life of drug-dependent patients. Only one previous systematic review included three studies [32] analyzing the quality of life in patients addicted to methamphetamine. To the best of our knowledge, no meta-analysis has been published that analyzes the quality of life in a drug-dependent population.

The results of our review, like those obtained by Morris et al. [32] in patients who met criteria for methamphetamine dependence, indicated an effect of exercise on quality of life, finding that eight articles out of 10 show significant improvements after participating in yoga programs [22,55,70,71], tai chi [73] or in aerobic and strength exercise programs [6,20,72]. In the same way, the results of the meta-analysis show a significant effect of the exercise on quality of life. This result is particularly important, both due to the lack of meta-analysis that address the study of the quality of life in the drug-dependent population, and the importance of improving the quality of life in these types of patients, since drug-dependent patients suffer from important deterioration of the quality of life, presenting significantly lower values than the healthy population [84].

In this sense, the data presented in this meta-analysis provides evidence for the use of physical exercise, especially programs that use combinations of aerobic exercise and strength and oriental practices, such as yoga or tai chi, for improving quality of life during the recovery process from drug dependency.

**Mental disorders**

The results obtained in mental disorders seem to show an effect of exercise on stress, anxiety and depression, although the results should be viewed in perspective. When analyzing the studies included in this review, it is observed that there are both studies that show significant improvements in stress, anxiety or depression, as studies that did not find significant improvements in these variables, or that these improvements were only intra-group, practically identifying the same number of studies in both senses. This fact could prevent us from drawing conclusions about the effectiveness of exercise in improving these variables. However, when analyzing in depth the studies that do not show significant improvements in stress, anxiety or depression, some methodological or structural aspects can be identified that could explain the non-achievement of statistically significant results, such as the short duration of the programs [53] and sparse samples [18,29,57,66]. Likewise, when observing the results of the meta-analysis, a significant effect of the exercise on mental disorders is identified, obtaining an average effect in anxiety and depression and a high effect in stress, for which, analyzing together the obtained data, it seems that positive effects of exercise on stress, anxiety and depression can be deduced.

The results obtained in anxiety and depression are consistent with those obtained in other reviews, where the beneficial role of exercise in these variables has been shown in patients who met criteria for methamphetamine dependence [32], AUD [34,81] or SUD [39]. It should be noted that the meta-analysis conducted by Hallgren et al. [34] found a significant effect of exercise on depression, but not on anxiety in AUD patients. Additionally, the review conducted by Giesen et al. [81] found a slight tendency to improve anxiety, but found no effect in depression in the same population. This contradiction in the benefits of exercise on anxiety and depression in AUD patients may be due to methodological irregularities and the disparity of measures used in some of the studies included in Giesen et al. or to the fact that only three studies were included in the meta-analysis conducted by Hallgren et al. Therefore, more randomized studies that assess the effect of exercise on anxiety and depression in this specific population and that allow meta-analysis with higher samples are required.

Assessing the results obtained in mental disorders in this meta-analysis highlights the effect of exercise on stress, finding a high effect of exercise on this variable. There are very few studies that have shown the effects of exercise on stress and no previous meta-analysis analyzing this variable in drug-dependent patients. In the present review, six experimental or quasi-experimental studies were identified that analyzed the effect of exercise on stress. Of these six, only two [15,55] showed statistically significant improvements compared to the control group after participating in two yoga programs. However, when observing the meta-analysis, more conclusive results are shown, identifying a great effect of the exercise on this variable. Once again, there is a need to carry out randomized studies with sufficiently representative samples and with structured exercise programs that rigorously allow the assessment of the effect of exercise on stress.

The management of stress in drug-dependent patients is crucial. This population has very high levels of stress, anxiety and depression [85]. In addition, stress is a variable directly related to the likelihood of relapse, increasing the patient’s vulnerability to relapse [86]. Therefore, improving stress through physical exercise could potentially exert a protective effect on patients and a boost to maintain abstinence.

**Craving**

The results of this study indicate a positive influence of exercise on craving, finding five studies that show significant improvements in craving after participation in exercise programs and only one [8] where improvements were not obtained in craving after participation in a four-week aerobic exercise program. In this study, perhaps the short duration of the program could have influenced the lack of improvements in craving. However, studies analyzing the acute effects of exercise on craving showed an improvement of craving after performing aerobic exercise, both in AUD patients [77] and in methamphetamine dependence patients [10,11]. Likewise, the results of the meta-analysis reinforce the conclusion that exercise can be a good way to reduce craving levels, finding a high effect of exercise on this variable. Previous review studies with AUD patients showed similar results [35,81], but to date, no known meta-analysis addressed this issue. Nevertheless, future studies should investigate the appropriate type and duration of exercise programs in order to maximize benefits in this regard.

The impulse and need to consume despite the serious consequences it entails, is one of the effects of drug-addiction [85]. These impulses are those that lead the drug-dependent patient to consume again and again before any other need. In periods of abstinence, the level of craving is directly related to the probability of having a relapse [87]. Therefore, to identify that physical exercise may reduce the levels of craving in this population is a very relevant result and gives physical exercise an important role in the rehabilitation of drug addictions, proving itself as an ideal means to increase the chances of success of the treatments.

**Abstinence/consumption**

The results of this review seem to indicate a certain benefit of the exercise on abstinence and drug use, finding numerous studies that show satisfactory results in abstinence or consumption after participating in exercise programs of different kinds. However, there are also several studies that did not show improvements in these variables in a significant way or that the improvements were only intra-group [8,16,29,52,59,60,69]. When analyzing the methodological quality and the possible biases in the studies, no substantial difference was found between the studies that showed statistically significant benefits on abstinence and those that did not. Therefore, no firm conclusions about the effect of exercise in the maintenance of abstinence can be drawn based on the systematic review. In similar lines, there were two reviews [35,81] and a meta-analysis performed in AUD patients [34], where they did not find a significant effect of exercise on alcohol consumption.

On the other hand, a meta-analysis conducted with SUD patients did draw firm conclusions in this field [39], stating that physical exercise can have an important effect for the maintenance of abstinence in tobacco, alcohol, and illicit drugs. The reason for this disparity in the results could be due to small sample sizes or unstructured exercise programs with a short duration or frequency.

Another reason that could influence the achievement or failure of significant results could be the adherence to the exercise programs. For example, in a randomized controlled study with a large sample [74], it was found that after the end of the exercise program there were no significant differences in abstinence compared to the control group. However, when performing the analysis controlling adherence to the program, they did find statistically significant improvements, concluding that mere participation in the program was not enough to achieve improvements in abstinence and that adherence to the exercise program was a determining factor in the achievement of the desired benefits. Therefore, future studies should investigate possible methodological strategies to increase adherence to exercise programs in this population, with the aim of increasing the effectiveness of the programs.

**Aerobic-strength exercise vs. Body–mind activities**

When analyzing the subcategories of mental disorders and quality of life, some differences can be observed. In quality of life, a greater effect of oriental practices was observed in the subcategories “physical function” and “pain”, while a greater effect of aerobic-strength exercise was shown in the variables “physical role”, “vitality” and “social function”. A similar effect on the variables “general health”, “emotional role” and “mental health” was observed.

In regards to mental disorders, oriental practices showed a greater effect on “anxiety” and “depression”, while aerobic-strength exercise shoed a greater effect on the variable “stress”. These results are not in agreement with those obtained by Wang et al. [39], where no differences were identified between both exercise practices in anxiety and depression. Notwithstanding, the results of the present study in this regard should be interpreted with caution, since when performing the analysis classifying by type of program, the number of included studies was lower. Furthermore, the number of studies included for each type of program was different, which may condition the results obtained.

Taking everything together, it can be deduced that both exercise practices are effective for the improvement of mental disorders and quality of life. Future meta-analysis studies that include more randomized studies of both practices and that control the bias that may exist at the time of performing the analysis, as well as randomized studies that compare the effects of each type of practice, should determine the suitability of applying one type of practice or another depending on the objectives of the treatment.

**Limitations and future studies**

This study presents a series of limitations, mainly due to the type of studies included. First, both randomized and nonrandomized studies were included. In addition, many of them presented methodological limitations, such as the non-performance of inter-group analysis, excessive number of dropouts, lack of control group or unrepresentative samples. These aspects may bias the results. However, it would also under represent the entire field of study.

In the same way, the high heterogeneity of the included studies can represent a bias when synthesizing the results. The selected studies include populations who met criteria of dependency for different substances, and different exercise programs with different structures in terms of duration, volume, intensity or frequency. Likewise, there are very different variables and measurement instruments and in some cases lack of relevant data. For example, many of the studies included did not report the severity of addiction prior to treatment.

All of this caused the loss of some studies at the time of performing the meta-analysis and may imply a bias in the research that must be taken into account.

On the other hand, of the 59 studies included in this research, 35 did not control adherence to the intervention program. However, adherence to exercise programs may determine the achievement of the intended objectives [74]. Drug-dependent patients have an average of dropouts in exercise programs much higher than dropouts registered in other special populations [34]. Not taking this issue into account can result in contradictory or misleading results, which can create confusion when drawing conclusions. In this way, control of adherence to exercise programs is especially important when performing research in this population.

Another methodological issue is the lack of information about the period of time that the benefits are maintained after the exercise intervention. Very few studies have included a follow up. In this sense, Sinyor et al. [50] showed that the benefits found in the abstinence of a sample of patients with AUD after an exercise program were maintained for 18 months after the end of the program. However, there is a clear need for more studies including follow-ups in order to draw firm conclusions about this issue.

There is currently no scientific evidence in regards to which specific physical capacities (endurance, strength, flexibility, balance, coordination) are more important from the point of view of the health and recovery of the drug-dependent patient. There is also no clear evidence about what volumes, intensities and frequencies are ideal for working with this population. Wang et al. [39] found that there was no difference between the application of exercise programs in terms of type of exercise and intensity, showing that mild and moderate intensities of the exercise achieved similar effects and that different types of exercise programs achieved similar effects. On the contrary, Wang et al. [11] concluded that a moderate or vigorous intensity of exercise supposed greater acute benefits in craving compared to a mild intensity after a single session of aerobic exercise. Therefore, there is still no clear evidence on the parameters of the exercise in terms of type of exercise, intensity, volume or frequency in this population. Thus, there is a need for randomized controlled studies with larger samples including structured exercise programs (detailing type, intensity, duration and frequency) and comparing different types, while controlling for the adherence to the program and including follow-ups, which will allow us to obtain reliable and comparable results, with the final aim of finding the optimal exercise prescription for this population.

## 5. Conclusions

This study shows relevant results about the benefits of exercise for health in drug-dependent patients.

First, it can be observed that aerobic physical exercise and combinations of aerobic exercise and strength are effective for the improvement of physical conditions in drug-dependent patients. Aerobic exercise programs are the most widely used in this population, although some studies combine aerobic and strength exercise, with satisfactory results.

One of the relevant results of this study is the significant effect of exercise on the quality of life in drug-dependent patients, with no previous meta-analysis addressing this issue. Similarly, it highlights the significant effect of exercise on stress, anxiety, depression and craving.

Despite the promising effects of physical exercise on abstinence, based on the results obtained in this study it cannot be concluded that there is solid evidence on this issue.

Finally, when comparing the effects of aerobic-strength exercise with oriental practices, no relevant differences were found about the overall value of quality of life and mental health. On the other hand, differences were observed in some of the subcategories of quality of life and mental health, although future studies must delve deeper into these issues to draw firm conclusions in this regard.

Thus, it can be concluded that aerobic physical exercise or combinations of aerobic exercise and strength are viable and effective practices for the improvement of physical conditions in this population. In addition, physical exercise, whether through pure aerobic exercise, combinations of aerobic exercise and strength or oriental practices such as yoga, tai chi or qigong, can be an effective way to improve stress, anxiety, depression, quality of life and craving in drug-dependent patients.

Therefore, it is recommended that physical exercise programs in rehabilitation centers are included in order to optimize the patient’s recovery process.

## Figures and Tables

**Table 1:** Methodological quality of the included studies. (PEDro Scale).

Article	1. Election Criteria	2. RTC	3. Blind Assignm.	4. Baseline Similar.	7. Blind Eval.	8. Measur. of >85%	9. Intent to Treat	10. Between Groups	11. Variab. Measures	Total
Gary et al. [46]	No	Yes	No	Yes	No	Yes	No	No	Yes	4/9
Frankel et al. [47]	No	No	No	No	No	No	No	No	Yes	1/9
Piorkowski et al. [48]	Yes	Yes	Yes	Yes	Yes	Yes	No	Yes	Yes	8/9
McKelvy et al. [49]	Yes	Yes	Yes	Yes	No	Yes	No	No	Yes	6/9
Sinyor et al. [50]	No	No	No	No	No	No	No	No	Yes	1/9
Palmer et al. [51]	Yes	No	No	Yes	No	No	No	Yes	No	3/9
Burling et al. [62]	Yes	No	No	Yes	No	No	No	Yes	No	3/9
Palmer et al. [63]	Yes	Yes	Yes	Yes	No	No	No	No	Yes	5/9
Donaghy et al. [52]	Yes	Yes	Yes	Yes	No	No	No	Yes	Yes	6/9
Ermalinski et al. [53]	No	No	No	No	No	No	No	Yes	No	1/9
Shaffer et al. [64]	Yes	Yes	Yes	Yes	No	No	No	Yes	No	5/9
Li et al. [65]	Yes	Yes	Yes	Yes	No	Yes	Yes	Yes	Yes	8/9
Ussher et al. [77]	Yes	Yes	No	Yes	No	Yes	Yes	Yes	Yes	7/9
Vedamurthachar et al. [54]	Yes	Yes	Yes	Yes	No	Yes	Yes	Yes	No	7/9
Sareen et al. [55]	Yes	Yes	Yes	Yes	No	Yes	Yes	No	No	6/9
Khalsa et al. [66]	Yes	No	No	No	No	No	No	No	Yes	2/9
Weinstock et al. [14]	Yes	No	No	No	No	Yes	No	Yes	Yes	4/9
Brown et al. [56]	Yes	No	No	No	No	No	No	No	Yes	2/9
Brown et al. [13]	Yes	No	No	No	No	No	No	No	Yes	2/9
Chen et al. [28]	No	No	No	Yes	No	No	No	Yes	No	2/9
Mamen et al. [26]	No	No	No	No	No	No	No	Yes	Yes	2/9
Roessler et al. [69]	Yes	No	No	No	No	No	No	No	No	1/9
Buchowski et al. [7]	Yes	No	No	No	No	Yes	No	No	Yes	3/9
Mamen et al. [16]	No	No	No	No	No	No	No	Yes	Yes	2/9
Dolezal et al. [68]	Yes	Yes	Yes	Yes	No	No	No	Yes	Yes	6/9
Li et al. [30]	Yes	Yes	Yes	No	No	No	No	Yes	Yes	5/9
Roessler et al. [57]	Yes	No	No	No	No	No	No	No	Yes	2/9
Smelson et al. [9]	Yes	Yes	Yes	No	No	Yes	No	Yes	Yes	6/9
Zhuang et al. [21]	Yes	Yes	Yes	Yes	No	Yes	No	Yes	Yes	7/9
Brown et al. [4]	Yes	Yes	Yes	Yes	No	Yes	Yes	Yes	Yes	8/9
Cutter et al. [69]	Yes	Yes	Yes	No	No	Yes	No	Yes	Yes	6/9
Devi et al. [70]	Yes	Yes	Yes	Yes	No	No	No	Yes	Yes	6/9
Dolezal et al. [27]	Yes	Yes	Yes	Yes	No	Yes	Yes	Yes	Yes	8/9
Flemmen et al. [18]	Yes	Yes	Yes	Yes	No	No	Yes	Yes	Yes	7/9
Hallgren et al. [29]	Yes	Yes	Yes	No	No	No	No	Yes	Yes	5/9
Jamurtas et al. [78]	No	No	No	No	No	Yes	Yes	Yes	Yes	4/9
Muller et al. [20]	Yes	No	No	No	No	No	No	Yes	No	2/9
Agarwal et al. [15]	Yes	Yes	Yes	Yes	No	No	No	No	Yes	5/9
Dhawan et al. [71]	Yes	Yes	No	Yes	No	No	No	Yes	Yes	5/9
Georgakouli et al. [79]	Yes	No	No	Yes	No	Yes	Yes	Yes	Yes	6/9
Giménez et al. [72]	Yes	No	No	Yes	No	No	No	Yes	Yes	4/9
Rawson et al. [17]	Yes	Yes	Yes	Yes	No	Yes	No	Yes	Yes	7/9
Wang et al. [10]	Yes	No	No	No	No	Yes	Yes	No	Yes	4/9
Brown et al. [5]	Yes	Yes	Yes	No	No	No	No	No	Yes	4/9
Ciccolo et al. [80]	Yes	No	No	No	No	No	No	No	Yes	2/9
Grandjean da Costa et al. [22]	Yes	No	No	No	No	No	No	No	Yes	2/9
De la Garza et al. [8]	Yes	Yes	Yes	Yes	No	Yes	Yes	Yes	Yes	8/9
Giesen et al. [6]	Yes	No	No	Yes	No	No	No	Yes	Yes	4/9
Wang et al. [11]	Yes	Yes	No	Yes	No	Yes	Yes	Yes	Yes	7/9
Zhu et al. [73]	Yes	No	No	No	No	Yes	No	Yes	Yes	4/9
Colledge et al. [25]	Yes	Yes	Yes	Yes	No	No	No	Yes	Yes	6/9
Georgakouli et al. [58]	Yes	No	No	No	No	No	No	No	Yes	2/9
Roessler et al. [59]	Yes	Yes	Yes	Yes	No	Yes	Yes	Yes	Yes	8/9
Trivedi et al. [74]	Yes	Yes	Yes	Yes	No	Yes	Yes	Yes	Yes	8/9
Wang et al. [12]	Yes	Yes	Yes	Yes	No	No	No	Yes	Yes	6/9
Carmody et al. [75]	Yes	Yes	Yes	Yes	No	No	Yes	Yes	Yes	7/9
Jensen et al. [60]	Yes	Yes	Yes	Yes	No	No	No	Yes	Yes	6/9
Roessler et al. [61]	Yes	Yes	Yes	Yes	No	Yes	No	Yes	No	6/9
Zhang et al. [76]	Yes	Yes	No	Yes	No	Yes	No	Yes	Yes	6/9

**Table 2:** Description of studies that measure the acute effect of exercise on alcohol or other drugs.

Article	Subst.	N (Exp + Control)	Program	Adherence (% Attendance)	Measuring Instruments	Main Results
Ussher et al. [77]	Alcohol	20 (All went through the exp/control conditions)	10 min of static bicycle. Experimental session: Moderate intensity (40–60% FC reserve) Control session: Very slight intensity (5–20% FC reserve)	-	Alcohol Urge Questionnaire Six-item measure of mood disturbance	Decrease in the impulse to consume alcohol during exercise in an experimental group compared to the control group. There were no significant differences at the end of the exercise.
Jamurtas et al. [78]	Alcohol	17 (9 + 8)	A mild intensity cycle ergometer session (55–60% FCM)	-	Alcohol Urge Questionnaire B-E Analysis Blood Measurements	Increase in B-endorphin levels There were no significant improvements Craving.
Georgakouli et al. [79]	Alcohol	34 (17 + 17)	A 30 min session of moderate intensity aerobic exercise (cycle ergometer)	-	Metabolic measurements	The response of liver enzymes was improved.
Wang et al. [10]	Methamphetamine	24 (All went through the exp/control conditions)	Experimental session: 30 min of static bicycle moderate intensity (65–75% FC max) Control session: 30 min of reading.	-	Visual Analog Scale for craving measurement Standard and MA-related Go/Nogo tasks Electroencephalogram	Improvements in craving during, immediately after and 50 min after exercise, with respect to the control group. Improvements at the cognitive level (concentration).
Brown et al. [5]	Alcohol	26	12 weeks aerobic exercise moderate intensity	70%	Likert scale (not validated) to measure mood and anxiety and craving.	Acute improvements after each exercise session in mood and anxiety.
Ciccolo et al. [80]	Alcohol	14 (All went through the exp/control conditions)	A session (20 min) of strength and a passive display (20 min) of video.	70%	Alcohol Use Disorders Identification Test (AUDIT) The Alcohol Urge Questionnaire Borg Rating of Perceived Exertion The Feeling Scale	Improvements in affection and arousal acutely. No significant improvements were found for craving.

**Table 3:** Studies that measure the effect of long-term exercise on alcohol use disorder (AUD) patients.

Article	Substance	N (Exp + Control)	Program	Adherence (% Attendanc.)	Measuring Instruments	Main Results
Gary et al. [46]	Alcohol	20 (10 + 10)	Experimental: 4 weeks, 5 sessions (run 1 mile per day) per week Control: Conventional treatment, group therapy.	-	Gough Adjective Check List Jourard Body-Cathexis Scale Self-Cathexis Scale Schneider Physical Test Control of alcohol consumption Control of sleep problems	Significant improvements at the cardiovascular level Lower level of sleep problems.
Frankel et al. [47]	Alcohol	214	Experimental: 12 weeks of aerobic exercise y strength, 5 sessions (60 min) per week.	-	Cardiovascular and anthropometric measures Illinois Standard Test of Physical Fitness Minnesota Multiphasic Personality Inventory	Significant improvements in physical conditions Significant improvements in some variables of the personality test.
Piorkowski et al. [48]	Alcohol	26 (14 + 12)	Experimental: 4 weeks of circuit training, 5 sessions (55 min) per week Control: table games, 5 sessions per week	-	Stair climb Cardiac frequency	Improvements in physical conditions.
McKelvy et al. [49]	Alcohol	48 (31 + 17)	Experimental: 4 weeks, 5 sessions (1,2 miles of running) per week at 85% FC max Control: Sedentary activities	-	One-Minute Step Test Three-Minute Step Test Manual capture of FC (carotid palpation)	Significant improvements in FC of rest and FC during effort.
Sinyor et al. [50]	Alcohol	79 (58 + 12 + 9)	Experimental: 6 weeks of aerobic exercise and stretching, 5 sessions (40–50 min) per week Control: Conventional treatment.	-	Cardio-physiological and anthropometric measurements	Greater abstinence from experimental group at the end of treatment and at 3 and 18 months of follow-up Fitness improvements
Palmer et al. [51]	Alcohol	53 (27 + 26)	Experimental: 4 weeks of aerobic exercise, 3 sessions (40–50 min) per week. Control: Conventional treatment	-	Zung Self-Rating Depression Scale Spielberger State-Trait Anxiety Inventory Tennessee Self-Concept Scale Astrand-Rhyming bicycle ergometer test	Improvements in anxiety and depression There were no improvements in physical conditions in experimental group.
Donaghy et al. [52]	Alcohol	158 (80 + 78)	Experimental: Aerobic and strength exercises. 15 weeks, 3 sessions (30 min) per week Control: Stretching and breathing exercises	-	Test of physical conditions (Sit and reach, sit up, VO2) Weight control Resting heart rate Physical Self-Perception Profile Beck depression inventory Zung Self-Rating Anxiety Scale	There were no differences in abstinence Improvements in physical conditions Improvements in anxiety and depression in both groups, without differences between groups.
Ermalinski et al. [53]	Alcohol	90 (48 + 42)	Experimental: 6 weeks of “body–mind-component” (yoga, jogging, motivational training) 5 sessions (90 min) per week. Control: Conventional treatment.	-	Multidimensional Health Locus of Control Scales Body cathexis scale Self-Cathexis scale Depression adjective check lists	Improvements in systolic blood pressure and aerobic capacity significantly Significant improvements in “craving” in experimental group Significant improvements in locus of control and responsibility in health care There were no improvements in depression or in “body satisfaction”.
Vedamurthachar et al. [54]	Alcohol	60 (30 + 30)	Experimental: 2 weeks of Sudarshana Kriya Yoga, 60 min of daily yoga Control: Conventional treatment	-	Beck Depression Inventory Analysis of cortisol and ACTH levels	Improvements in depression in both groups, but significantly more in the experimental group Decrease of ACTH and cortisol, but significantly more in experimental group.
Sareen et al. [55]	Alcohol	52 (26 + 26)	12 weeks of yoga, 3 sessions (60 min) per week Control: Conventional treatment	-	Short Form Health Survey (SF-36) The Profile of Mood States (POMS) The Symptoms of Stress Inventory (SOSI)	Significant improvements in quality of life, stress, mood, alcohol dependence and appetite.
Brown et al. [56]	Alcohol	19	12 weeks of aerobic exercise moderate intensity (1 day per week supervised, the rest autonomous) and 1 day weekly of teaching proper physical-sports habits.	68% (to supervised sessions) Economic incentives to favor adherence	Structured Clinical Interview for DSM-IV Timeline Follow Back Expired air analysis Submaximal effort test Cutaneous folds and scale meter	Abstinence improvements Improvements in cardiorespiratory fitness Decrease in BMI.
Roessler et al. [57]	Alcohol	7	6 weeks of aerobic work, moderate intensity, 2 sessions per week	59%	Bruce treadmill protocol Screening Questionnaire of Common Mental Disorders Becks Depression Inventory	There were no significant improvements in physical conditions. A tendency was observed in the improvement of anxiety, depression and emotional disorders, but without significant results.
Brown et al. [4]	Alcohol	49 (26 + 23)	12 weeks of moderate intensity aerobic exercise (1 day per week supervised, the rest autonomous) and 1 day weekly of teaching proper physical-sports habits.	70% (to supervised sessions) Economic incentives to favor adherence	Timeline Follow Back Analysis of expired air Center for Epidemiological Studies-Depression scale The Spielberger State-Trait Anxiety Inventory Situational Confidence Questionnaire Submaximal effort test	Significant improvements in abstinence/alcohol consumption Better results in abstinence/consumption in participants who attended more than 66% of the sessions There were no significant differences in VO2, anxiety, depression and self-efficacy.
Hallgren et al. [29]	Alcohol	14 (6 + 8)	Experimental: 10 weeks of yoga, 1 session (90 min) weekly group. Patients were encouraged to perform yoga exercises autonomously. Control group: Conventional treatment.	-	Timeline Follow-back Method DSM-IV criteria for alcohol dependence Short Alcohol Dependence Data Questionnaire Hospital Anxiety and Depression Scale Sheehan Disability Scale Perceived Stress Scale	Quantitative: There were no significant differences in any variable Qualitative: Improvements in perception, stress and anxiety, increase in well-being, improvement of sleep quality, improvements in craving.
Brown et al. [5]	Alcohol	26	12 weeks of moderate intensity aerobic exercise	70%	Likert scale (not validated) to measure mood and anxiety and craving.	Acute improvements in mood and anxiety.
Giesen et al. [6]	Alcohol	33 (14 + 19)	Experimental: 12 months, 2 sessions (60 min) per week. Example: Aerobic, strength, coordination and flexibility. Control: Conventional treatment	-	Daily monitoring of physical activity levels Short Form Health Survey (SF-36) Toxicological test	Significant improvements in quality of life (variables physical function, vitality, emotional role and mental health).
Georgakouli et al. [58]	Alcohol	20 (9 + 11)	8 weeks of supervised exercise	-	Anthropometric and physiological measurements International Physical Activity Questionnaire Alcohol Use Disorders Identification Test (AUDIT)	Improvements in alcohol consumption and physical conditions.
Roessler et al. [59]	Alcohol	172 (61 + 59 + 52)	6 months of aerobic exercise, mild-moderate intensity, 2 sessions (30-60 min) per week Exp. group 1: in groups Exp. group 2: Individual Control: Conventional treatment	-	The Addiction Severity Index The Timeline Follow-Back questionnaire International Physical Activity Questionnaire	There was no effect of the exercise program on alcohol consumption A greater protective effect of excessive alcohol consumption was observed in patients with a higher level of physical activity.
Jensen et al. [60]	Alcohol	105 (38 + 35 + 32)	24 weeks of aerobic exercise, 2 sessions (30-60 min) per week Exp. group 1: in groups Exp. group 2: Individual Control: Conventional treatment	-	The Bruce treadmill protocol Timeline Follow Back Measures of VO2 and FC	Only the group that worked individually improved their VO2, but without significant differences between groups. There were no significant differences in alcohol consumption between groups.
Roessler et al. [61]	Alcohol	116 (81 + 35)	6 months of aerobic exercise, mild-moderate intensity, 2 sessions (30–60 min) per week. Exp. group 1: in groups Exp. group 2: Individual Control: Conventional treatment	-	The Inventory of Interpersonal Problems	There were no significant changes between the control and experimental groups in any of the 4 measured subscales.

**Table 4:** Studies that measure the effect of long-term exercise on substance use disorder (SUD) patients.

Article	Population/Substance	N (Exp + Control)	Program	Adherence (%Attendance)	Measuring Instruments	Main Results
Burling et al. [62]	SUD (varied substances)	95 (34 + 61)	Exp group: 4 weeks of softball, 1 session per week Control group: Conventional treatment	-	Abstinence Sociodemographic data	Improvement in abstinence.
Palmer et al. [63]	SUD (varied substances)	45	4 weeks of supervised exercise, 3 sessions (30–40 min): G. 1: Aerobic 60% FC max G. 2: Body-building G. 3: Circuit training	-	Health Status Questionnaire Center of Epidemiological Studies-Depression Kasch Step Test Anthropometric and cardio-physiological measurements	Physical condition did not improve in any group Significant improvements in depression in group 2 (body building).
Shaffer et al. [64]	SUD in maintenance with methadone	59 (30 + 29)	Exp. group: 22 weeks of yoga, 75min per session Control group: Psychotherapy	-	Symptom check list Addiction severity index	There were not significant differences in any variable between the two groups.
Li et al. [65]	Heroin	86 (34 + 26 + 26)	Exp. group: 10 days of qigong, 25–30 min per session, without pharmacological treatment Control group 1 and 2: different pharmacological treatments, without physical exercise.	100%	Urine analysis Electrocardiogram Hamilton Anxiety Scale Withdrawal-symptom evaluation scale	Significant improvement with respect to the control groups in symptoms of withdrawal syndrome, anxiety, and consumption.
Khalsa et al. [66]	SUD (varied substances)	8	90 days of yoga	-	The Perceived Stress Scale (PSS) The 32-item Behavior and Symptom Identification Scale (BASIS-32) Quality of Recovery Index (QRI)	Improvement in the BASIS-32 and QRI test. There was a decrease in perceived stress (PSS), but it was not significant.
Weinstock et al. [14]	SUD (varied substances)	187 (45 + 142)	G. exp: Chose at least one sport activity offered. Control group: Did not do any sports activity.	-	Addiction Severity Index Urine and air test	Improvements in abstinence in participants who did physical-sports activities.
Brown et al. [13]	SUD (varied substances)	16	12 weeks of moderate intensity aerobic exercise (1 day weekly supervised, the rest autonomous work) and 1 day weekly of teaching proper physical-sporting habits.	71% (supervised sessions) Economic incentives to favor adherence	Structured Clinical Interview for DSM-IV Timeline Follow Back Expired air analysis Submaximal effort test Folds meter	Abstinence improvements Improvements in cardiorespiratory fitness There were no differences in body composition.
Chen et al. [28]	SUD (varied substances)	207 (126 + 81)	G. exp: qigong G. control: Standard relaxation techniques (SMART) One session daily of qigong (1st phase) or two sessions daily (2nd phase) of qigong or SMART during 4 weeks.	Experimental group: 92% Control group: 78%	Adjectuve Rating Scale for Withdrawal Voris craving/negative-mood scale CES Depression Scale Spielberger State–Trait Anxiety Inventory–State only Substance-specific craving scale	Both groups improved in craving, sleep, anxiety, depression and symptoms of withdrawal syndrome, without significant differences between groups, except in craving, where qigong Group improved more than SMART group. Qigong Group achieved greater adherence to the program.
Mamen et al. [26]	SUD (varied substances)	33	7.5 months (300 h) of aerobic exercise (light intensity) individualized, with “trainer partners”	-	Lactate measurements Heart rate 15-point rating of perceived exertion Maximum VO2 test	Significant improvements in aerobic power and production/elimination of lactate.
Roessler et al. [67]	SUD (varied substances)	20	2–6 months of aerobic exercise, 3 sessions (120 min) per week.	52%	Indirect VO2 maximum test (www.steptest.dk) European Addiction Severity Index 19 Semi-structured interviews	Quantitative: Significant improvements in VO2 maximum Qualitative: Improvements in perception of quality of life, energy level, body image and decrease in consumption.
Buchowski et al. [7]	Cannabis (active consumers)	12	10 sessions (30 min) of moderate intensity supervised aerobic exercise.	100%	Marijuana Craving Questionnaire Timeline Follow-back Method	Less cannabis use during the program Craving improvements.
Mamen et al. [16]	SUD (varied substances)	33	Between 2–15 months (average duration: 7.5 months, 301 h) of aerobic exercise (mild intensity) individualized	-	Lactate measurements VO2 max test The Beck Depression Inventory The Beck Anxiety Inventory The Brief Social Phobia Scale The Symptom Checklist-90 The Short Michigan Alcoholism Screening Test The Drug Abuse Screening Test-20	Significant improvements in aerobic power and production/elimination of lactate. Decreased levels of anxiety, depression, social phobia and mental anguish. No significant improvements in alcoholism and drug abuse tests.
Dolezal et al. [68]	Methamphetamine	29 (15 + 14)	8 weeks, 3 days (60 min) per week. Experimental group: aerobic endurance and muscular strength Control group: Educational intervention, without exercise.	Experimental group: 92% Control group: 96% Economic incentives	Scale, stadiometer and bending meter for anthropometric measurements Stress test to measure VO2 and cardiac function Electrocardiogram 1RM maximum leg press and pectoral press	Significant improvements in VO2 and strength of legs and chest. Significant decrease in body fat and total weight Non-significant increase in fat-free weight.
Li et al. [30]	Heroine (women)	33 (17 + 16)	Experimental group: 6 months of tai chi, 2–3 sessions (60 min) per week. Control group: Conventional treatment	-	Blood test Hamilton Rating Scale for Depression Rating scale of heroin withdrawal symptoms	At the end of the program, there were no statistically significant differences between the two groups in any of the variables measured.
Smelson et al. [9]	Cocaine	86 (45 + 41)	2 weeks of qigong, between 4–6 sessions. Experimental group: real qigong Control group: simulated qigong.	Experimental group: 90% Control group: 74%	Cocaine Craving Questionnaire Brief Voris Cocaine Craving Scale Spielberger State-Trait Anxiety Inventory– State only Beck Depression Inventory Credibility/ Expectancy Questionnaire Addiction Severity Index	Improvements in craving and depression.
Zhuang et al. [21]	Heroine (women)	75 (37 + 38)	Experimental group: 6 months of yoga, 5 sessions (50 min) per week. Control group: Conventional treatment	100%	The Profile of Mood States (POMS) Short Form Health Survey (SF-36)	Improvements in quality of life (variables with physical role, pain, general health, vitality, emotional role and mental health). Improvements in mood in the variables tension and anxiety, depression, fatigue and confusion.
Cutter et al. [69]	Cocaine and/or opiates in maintenance with methadone	27 (14 + 13?)	8 weeks, 5 sessions (20–25 min) per week. Experimental group: Physical exercise (aerobic, strength, balance and yoga) through video game (Wii Fit Plus) Control Group: Sedentary video game (Wii).	Experimental group: 63% Control group: 68%	International Physical Activity Questionnaire-Long Version (IPAQ) Toxicological analysis of urine Timeline Follow Back. Perceived Stress Scale Life Orientation Test-Revised Brief Symptom Inventory-18 Brief Life Satisfaction Scale	The experimental group showed higher levels of physical activity in their day to day (IPAQ) The two groups improved in drug use, optimism and perceived stress, without differences between groups. There were no significant differences in global psychiatric symptoms or in life satisfaction.
Devi et al. [70]	SUD (various substances)	66 (33 + 33)	Experimental group: 4 weeks of yoga, 70 min daily Control group: Conventional treatment	-	Beck Depression Inventory WHO Quality of Life –BREF	Significant improvements in depression and quality of life (domains physical health, psychological health and social relationships).
Dolezal et al. [27]	Methamphetamine	28 (14 + 14)	8 weeks, 3 days (60 min) per week. Experimental group: aerobic endurance and muscular strength Control group: Educational intervention, without exercise.	Experimental group: 92% Control group: 96% Economic incentives to favor adherence.	Scale, stadiometer and bending meter for anthropometric measurements Stress test to measure VO2 and cardiac function Electrocardiogram 1RM maximum leg press and chest press	Significant improvements in VO2 and strength of legs and chest Improvements in body composition Significant improvements in heart rate variability rates.
Flemmen et al. [18]	SUD (various substances)	16 (9 + 7)	Experimental group: 8 weeks, 3 days per week of high intensity interval training (4 × 4 ‘90–95% HRmax) Control group: Conventional rehabilitation treatment.	92%	Stress test (Cortex Metamax II portable metabolic test system) to assess VO2 and effort economy. Addiction Severity Index Insomnia Severity Index Hospital Anxiety and Depression Scale	Significant improvement in VO2 Significant improvement (intragroup, but not between groups) in depression There were no significant differences in effort economy, insomnia and anxiety.
Muller et al. [20]	SUD (various substances)	35 (24 + 11)	Experimental group: 10 weeks of aerobic exercise and light strength, 3 sessions (30 min) per week. Control group: Conventional treatment	69%	The World Health Organization Quality of Life Brief The Hopkins Symptoms Checklist European Addiction Severity Index	Significant improvements in the domains “Physical Health” and “Psychological Health” of Quality of Life. Improvements in anxiety and depression, but not significant.
Agarwal et al. [15]	Consumers of crack with HIV (cocaine)	24 (12 + 12)	Experimental: 8 weeks of yoga/Meditation, 2 sessions (60 min) per week. Control: Conventional treatment	88%	Short Form 36 Health Survey (SF-36) Perceived Stress Scale (PES) Impact of Events Scale(IES) Measurements of cortisol and DHEA-S in saliva	Significant improvements in perceived stress (PES and IES) No changes in Cortisol and DHEA-S in saliva. There were no significant improvements in quality of life.
Dhawan et al. [71]	Opiates (60% of heroin sample)	84 (55 + 29)	G. exp: 3 sessions (60 min) of yoga. G. control: Not specified	-	World Health Organization quality of life brief scale Urine toxicology test	Improvements in quality of life in the domains of physical health, psychological health and the environment.
Giménez et al. [72]	SUD (various substances)	37 (18 + 19)	Experimental group: 12 weeks, 3 sessions (60–90 min) per week of aerobic endurance, strength-endurance and aerobic games, moderate intensity, Control group: Conventional treatment.	81%	Six-Minute Walk Test (6MWT) Timed Get Up and Go Test (TGUG) Chair Stand Test (CST) Short Form Health Survey (SF-36) 11 Semi-structured interviews	Quantitative: Significant improvements in physical conditions and quality of life. Qualitative: Decrease in the number of injuries when exercising, weight loss, improvements in stress and anxiety, improvements in craving.
Rawson et al. [17]	Methamphetamine	135 (69 + 66)	Experimental group: 8 weeks of aerobic exercise (60–80% HRmax) and strength, 3 sessions (60 min) per week. Control group: Education for health	Experimental group: 72% Control group: 77% Economic incentives	The Beck Depression Inventory The Beck Anxiety Inventory	Significant improvements in anxiety and depression Positive relationship between adherence to the program and the benefits obtained in anxiety and depression.
Grandjean da Costa et al. [22]	Crack and cocaine	9	12 weeks aerobic exercise (running), 3 sessions (60 min) per week	62%	Cooper 12-min test PAR-Q (Physical Activity Readiness Questionnaire) Blood pressure measurements Heart rate	Significant improvements at the cardiovascular and cognitive levels.
De la Garza et al. [8]	Cocaine	24 (10 + 7 + 7)	4 weeks, 3 sessions (30 min)/week: G. exp. 1: 30 min of walking on belt (25% of Maximum Heart Rate) G. exp. 2: 30 min of running on belt (75% of Maximum Heart Rate) G. control: sedentary activity	-	Heart rate Urine tests to identify cocaine use Subjective measurement measures of “craving”	Significant differences between the “runners” and “sedentary” groups in FC rest. There were improvements in abstinence in the experimental groups, but not statistically significant.
Zhu et al. [73]	Methamphetamine	59 (30 + 29)	G. exp: 12 weeks of Tai chi, 5 sessions per week G. control: Conventional treatment, recreational activities	-	Quality of life for drug addiction (QOL-DA) Anthropometric measures Sit and reach test	Improvements in quality of life in the Tai chi group compared to the control group.
Colledge et al. [25]	Heroine	24 (11 + 13)	Experimental group: 12 weeks of varied exercise, two sessions per week Control group: Not specified	-	German version of the Centre for Epidemiologic Studies Depression Scale Self-report Insomnia Severity Index (ISI) Brief Self Control Scale (BSCS) Perceived Stress Scale (PSS) Short-form health survey questionnaire (SF-36) Timeline Follow-back (TLFB) Physical Activity Questionnaire Short Form	The exercise group increased its daily exercise levels significantly, but no significant improvement was observed in any of the other variables measured.
Trivedi et al. [74]	Stimulants	302 (152+150)	Experimental group: 12 weeks of aerobic exercise, three sessions per week. Control group: Education for health	Experimental group: 64% Control group: 74.7%	Timeline Follow Back Urine analysis	No significant differences between groups, but when controlling adherence, a significant improvement in abstinence was detected
Wang et al. [12]	Methamphetamine	50 (25 + 25)	G. exp: 12 weeks, 3 sessions (30 min) per week of moderate intensity aerobic exercise and behavioral treatment. G. control: Conventional treatment and behavioral treatment	-	Visual analogue scale for craving measurement Standard Go/Nogo and MA-related Go/Nogo tasks Electroencephalogram	Significant improvements in craving and self-control compared to control group.
Carmody et al. [75]	Stimulant drugs (cocaine, amphetamine, methamphetamine)	218 (75 + 143)	Experimental group: 9 months of aerobic exercise, moderate-vigorous intensity, three sessions per week Control group: Health education	-	Timeline Follow Back Stimulant Selective Severity Assessment Concise Associated Symptoms Tracking- Self-Report Addiction Severity Index	The experimental group presented significantly lower probability of relapse and lower consumption in case of having a relapse than the control group.
Zhang et al. [76]	Methamphetamine	66 (34 + 32)	G. exp: 12 weeks, 3 sessions (30 min) per week of ex. aerobic moderate intensity G. control: Conventional treatment	-	CogState battery assessment Blood samples	The experimental group improved the speed of information processing and fat oxidation.
