# Correction: Giménez-Meseguer, J., Tortosa-Martínez, J., Cortell-Tormo, J. M. The Benefits of Physical Exercise on Mental Disorders and Quality of Life in Substance Use Disorders Patients. Systematic Review and Meta-Analysis. *International Journal of Environment Research and Public Health.* 2020, *17*, 3680

**DOI:** 10.3390/ijerph17145184

**Published:** 2020-07-17

**Authors:** Jorge Giménez-Meseguer, Juan Tortosa-Martínez, Juan M. Cortell-Tormo

**Affiliations:** Faculty of Education, University of Alicante, 03690 San Vicente del Raspeig, Spain; jgimenezmeseguer@gmail.com (J.G.-M.); jm.cortell@gcloud.ua.es (J.M.C.-T.)

The authors wish to make the following corrections to this paper [1]:

On page 1, in the abstract, lines 17–22, the paragraph “An effect of exercise on mental disorders (standardized mean differences (SMD) = 0.66 (confidence interval (CI): 0.46, 0.86); z = 6.50; *p* < 0.00001) and quality of life (SMD = 0.69 (95% CI: 0.53, 0.84); z = 8.65; *p* < 0.00001) was identified. Subgroup analysis revealed an effect of exercise in craving (SMD = 0.80 (CI: 0.07, 1.53); z = 2.15, *p* = 0.03), stress (SMD = 1.11 (CI: 0.31, 1.91); z = 2.73; *p* = 0.006), anxiety (SMD = 0.50 (CI: 0.16, 0.84); z = 2.88; *p* = 0.004) and depression (SMD = 0.63 (CI: 0.34, 0.92); z = 4.31; *p* < 0.0001)” should be “An effect of exercise on quality of life and mental disorders was identified. Subgroup analysis revealed an effect of exercise on stress (SMD = 1.11 (CI: 0.31, 1.91); z = 2.73; *p* = 0.006), anxiety (SMD = 0.50 (CI: 0.16, 0.84); z = 2.88; *p* = 0.004) and depression (SMD = 0.63 (CI: 0.34, 0.92); z = 4.31; *p* < 0.0001), and an effect of exercise on the eight variables included in the SF36 test. The results also showed a trend towards a positive effect on craving (SMD = 0.89 (CI: -0.05, 1.82); z = 1.85, *p* = 0.06)”.

On page 23, lines 6–7, the sentence “The results show also a significant effect of exercise on craving (k = 3; SMD = 0.80 (CI: 0.07, 1.53); z = 2.15; *p* = 0.03)” should be “The results also show a trend towards a positive effect of exercise on craving (k = 3; SMD = 0.89 (CI: −0.05, 1.82); z = 1.85; *p* = 0.06)”.

On page 29, lines 21–23, the sentence “Likewise, the results of the meta-analysis reinforce the conclusion that exercise can be a good way to reduce craving levels, finding a high effect of exercise on this variable” should be “The results of the meta-analysis show also a trend towards a positive effect of exercise on craving”.

The authors would like to apologize for any inconvenience caused to the readers by these changes.
